# Herpesviral Latency—Common Themes

**DOI:** 10.3390/pathogens9020125

**Published:** 2020-02-15

**Authors:** Magdalena Weidner-Glunde, Ewa Kruminis-Kaszkiel, Mamata Savanagouder

**Affiliations:** Department of Reproductive Immunology and Pathology, Institute of Animal Reproduction and Food Research of Polish Academy of Sciences, Tuwima Str. 10, 10-748 Olsztyn, Poland; e.kruminis-kaszkiel@pan.olsztyn.pl (E.K.-K.); mamata.savanagouder@pan.olsztyn.pl (M.S.)

**Keywords:** herpesvirus, latency, reactivation, gene expression, lytic cycle, persistence, lytic inducers, Alphaherpesvirinae, Betaherpesvirinae, Gammaherpesvirinae

## Abstract

Latency establishment is the hallmark feature of herpesviruses, a group of viruses, of which nine are known to infect humans. They have co-evolved alongside their hosts, and mastered manipulation of cellular pathways and tweaking various processes to their advantage. As a result, they are very well adapted to persistence. The members of the three subfamilies belonging to the family Herpesviridae differ with regard to cell tropism, target cells for the latent reservoir, and characteristics of the infection. The mechanisms governing the latent state also seem quite different. Our knowledge about latency is most complete for the gammaherpesviruses due to previously missing adequate latency models for the alpha and beta-herpesviruses. Nevertheless, with advances in cell biology and the availability of appropriate cell-culture and animal models, the common features of the latency in the different subfamilies began to emerge. Three criteria have been set forth to define latency and differentiate it from persistent or abortive infection: 1) persistence of the viral genome, 2) limited viral gene expression with no viral particle production, and 3) the ability to reactivate to a lytic cycle. This review discusses these criteria for each of the subfamilies and highlights the common strategies adopted by herpesviruses to establish latency.

## 1. Introduction

Herpesviruses are a family of enveloped viruses with large double-stranded DNA genomes [1]. Their life cycle is characterized by two phases: the lytic cycle—when the virus is actively replicating and the latency, which enables these viruses to persist for the lifetime of the host. Latency is defined as a state in which: 1—the viral genome persists; 2—viral gene expression is limited and there is no viral particle production, and 3—there is a possibility of reactivation back to the lytic replication [2]. These three criteria set latency apart from a persistent infection, during which new viral particles are produced; and abortive infection, in which the virus is unable to reactivate [1]. The name of this virus family derives from the Greek word herpes (to creep) and originally referred to the spreading lesions observed during herpes simplex virus (HSV) or varicella zoster virus (VZV) infection [3], but it could also refer to the quiescent nature of herpesviral infections, since latency is the hallmark of their life cycle. 

Herpesviruses include three subfamilies: Alpha-, Beta-, and Gammaherpesvirinae, each one of them having different characteristics with regard to tropism, length of the replicative cycle and associated diseases. The Alphaherpesvirinae, including HSV-1 and 2 and VZV, show relatively broad host and cell tropism infecting both human and non-human cells. With regard to the cell types, in vitro virtually all types can become infected including fibroblasts, epithelial cells, and neurons [4]. The replicative cycle of alpha-herpesviruses is very short (hours), they spread fast in culture and latency is established in sensory neurons [5]. The Betaherpesvirinae include the human cytomegalovirus (HCMV), human herpesvirus 6A and B (HHV-6A and B), and human herpesvirus 7 (HHV-7) and have species-specific tropism and a longer (days) replication cycle [6]. They spread slower and the infection results in characteristic cell enlargement [3,7]. This subfamily infects epithelial cells, endothelial cells, smooth muscle cells, monocytes, T lymphocytes, and fibroblasts and establishes latency in CD34+ hematopoietic stem cells and CD14+ monocytes or T lymphocytes, but recent studies suggest that HCMV might also be able to persist in neuronal cells [3,7,8,9,10]. The Gammaherpesvirinae, which include Epstein–Barr virus (EBV) and Kaposi’s sarcoma-associated herpesvirus (KSHV), have very narrow tropism both with regard to the host and the cell type, they are species-specific and infect lymphocytes, epithelial and endothelial cells, and establish latency in B lymphocytes. Concerning comparison of the life cycles of the three herpesvirus subfamilies, it is important to state that for the alpha- and the beta-herpesviruses the lytic cycle is the default path, while for the gammaherpesviruses it is the latency that predominates. Consequently, the latency of the Gammaherpesvirinae has been studied the most and is therefore best understood. One more unique feature of the latent gammaherpesviruses is their association with oncogenesis. EBV is associated with large number of malignancies, among others Burkitt’s lymphoma, Hodgkin’s disease, nasopharyngeal carcinoma, and post-transplant lymphoproliferative disease (PTLD) [11]. KSHV is the causative agent of Kaposi sarcoma (KS), plasmablastic variant of Multicentric Castleman’s disease (MCD) and primary effusion lymphoma (PEL) [12]. Both of the viruses induce tumor formation through persistence of the viral genome and therefore continuous expression of viral latent proteins (see also below), which have the ability to inhibit apoptosis, evade anti-viral immune response, and induce proliferation [13]. 

## 2. Latent Genome Persistence

As mentioned earlier, herpesviruses have large dsDNA genomes that are linear when inside of the viral particle, however they become circularized upon entering the infected cell [1]. During the lytic phase herpesviruses use the ‘rolling circle mechanism’ to replicate their genome and most of the proteins needed for this process are encoded in the viral genomes. Lytically replicating virus practically converts the infected cell into ‘a virus-producing factory’, redirecting all the cellular processes towards viral protein and DNA synthesis. In contrast, during latency there is no particle production and the viral gene expression is limited to a minimum. Beta and gamma herpesviruses had to develop a strategy to maintain their latent genomes in dividing cells (hematopoietic progenitors or lymphoid cells, respectively). The genomes persist in the form of circular molecules—episomes, attached to cellular chromosomes with the help of viral and cellular proteins [1,14,15]. Alphaherpesviruses, on the other hand, establish latency in terminally differentiated, non-dividing neurons, therefore in this case, there is no risk of the viral genome being lost and consequently also no need to tether the episome to cellular chromatin. Among the human herpesviruses an exception with regard to the maintenance of the latent genome as an episome is HHV-6, which integrates in the telomere sequences of the host chromosomes [16,17,18,19,20,21]. The herpesviruses that establish latency in proliferating cells duplicate their latent episomes in the nucleus once per cell cycle, in synchronization with the cellular replication in the S phase [22]. In contrast to lytic replication that is executed mostly by the viral proteins, latent replication is performed with the help of cellular machinery. Additionally, gammaherpesviruses encode origin binding proteins (OBPs), latency-associated nuclear antigen 1 (LANA1) for KSHV and Epstein–Barr nuclear antigen 1 (EBNA1) for EBV, that are responsible for recruiting the cellular replication proteins to viral genomes [23,24,25,26]. Viral OBPs bind specific DNA sequences within the viral genome (latent origin of replication) and at the same time interact with cellular chromatin binding proteins, thus mediating the tethering of viral genomes to cellular chromosomes [26,27]. Even though both the beta- and the gammaherpesviruses persist in dividing cells, for a long time the OBPs have been known only for the gammaherpesviruses. However, recently, the existence of a latency protein responsible for both genome tethering and latent replication has been reported for HCMV [28]. Future research will show how similar in its functions is this protein to gammaherpesviral OBPs. 

Herpesviral genomes inside the viral particle are compacted with the help of spermine, but do not contain nucleosomes [29,30,31]. In contrast, latent genomes of herpesviruses are fully chromatinized, in a manner similar to the cellular DNA [32,33,34,35,36]. As a consequence, transcriptional activity of the viral latent chromatin can be regulated by histone modifications and their changes, using mechanisms as in the case of cellular chromatin.

## 3. Viral Gene Expression Patterns in Latency 

The original definition of latent and lytic states in case of herpesviruses describes the lytic cycle as the phase in which transcription from the viral genomes is very high, all or almost all the viral genes are expressed, and latency as a condition when only a limited number of genes is expressed. However, the more sensitive assays we use and the more we know about the latent expression the less limited it appears to be. The subfamily of herpesviruses that is most studied with respect to latency are the gammaherpesviruses; therefore, we will start the overview of the latent programs from them.

**Figure 1:**
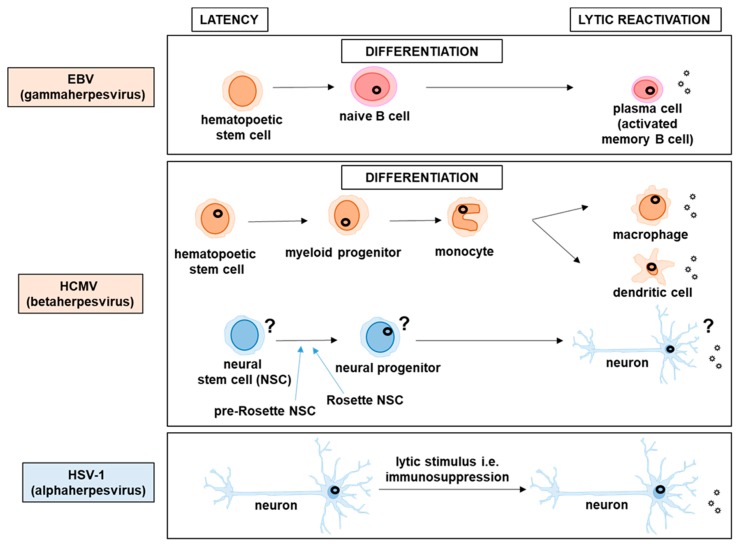
Latency establishment and lytic reactivation of herpesviruses. Following an initial lytic infection, herpesviruses establish latency mostly in dividing cells (except alphaherpesviruses), during which the viral genome is maintained as an episome and is tethered to cellular chromatin; lytic reactivation of gammaherpesviruses and betaherpesviruses may be cell differentiation dependent and occurs upon receiving the stimulus for terminal differentiation. EBV, a gammaherpesvirus, infects naïve B cells and remains latent throughout their differentiation until memory B cells carrying latent virus become activated and differentiate into plasma cells, which induces lytic reactivation. HCMV, a betaherpesvirus, establishes latency in cells of the myeloid lineage, its lytic cycle is activated upon differentiation into macrophages and dendritic cells. Additionally, HCMV can also establish latency in neural lineage and undergo lytic cycle in neurons, although the details of HCMV neural infection need to be studied further to resolve observed contradictions. In contrast HSV-1, an alphaherpesvirus establishes latency in sensory neurons and the lytic reactivation is induced upon receiving a specific stimulus. Viral genomes are symbolized by a black circle in the nucleus.

EBV infects naïve B cells, which are the most abundant cells circulating in the blood, and uses B cell differentiation to promote viral latency establishment in the long-lived memory B cells (Figure 1). Studies of EBV-induced tumors (Burkitt lymphoma, nasopharyngeal carcinoma, EBV positive Hodgkins disease) revealed that the virus engages different latency programs in different tumors [26]. Similarly, in the B cells of healthy EBV positive individuals, different viral transcription programs are found to be associated with specific differentiation stages of the B cell [37]. The EBV latency proteins include a set of EBNAs and three latency-associated membrane proteins (LMPs) (Table 1) [26]. The latency program with the highest expression is the type III latency found in the lymphoblastoid cell lines immortalized by EBV and the proteins expressed in these cells include: EBNA-1, -2, -3a, -3c, -3b, –LP, and LMP-1, -2a, and -2b [26]. This program is also found in infected naïve B cells [37]. In nasopharyngeal carcinoma and EBV positive Hodgkins disease the type II latency was detected, which is characterized by expression of EBNA-1 and LMP-1 and/or -2 [26]. Type II latency program was also found in germinal center B cells [37]. Cells of Burkitt lymphoma represent EBV type I latency expressing only EBNA1 [26]. This program is found also in proliferating memory B cells [37]. The quiescent memory B cells show the so-called type 0 latency, where only the viral genome is detected, but not the viral proteins. Finally, when the memory B cells become activated and turn into plasma cells, EBV enters the lytic cycle [37]. 

The functions of the EBV latent proteins allow the virus to successfully persist in the infected cells. EBNA-LP and EBNA-2 are responsible for the transcriptional regulation of expression of other latency proteins [38]. EBNA1 plays an important role in virus persistence, by tethering the viral genome to cellular chromosomes and participating in viral genome segregation and latent replication [39,40]. Additionally, EBNA1 acts also as a transcriptional activator [41,42]. LMP-1 belongs to the tumor necrosis factor (TNF) superfamily of proteins and mimics the CD4+ T cell signals. It promotes B cell survival by constitutively upregulating NF-κB signaling [43,44]. The membrane protein LMP-2a mimics B cell receptor survival signals [45]. 

In addition to protein expression, also non-coding RNAs are expressed in all types of EBV latency, these include EBV-encoded small RNAs (EBER 1 and EBER2), miRNAs encoded in the BamHI A rightward transcript (BART) and the BHRF1 locus as well as recently identified circular RNAs [46,47,48,49]. EBERs confer oncogenic properties on the virus infected cells and antagonize the function of PKR (protein kinase RNA-dependent), thereby preventing apoptosis of the infected cells and influencing the innate immune responses [50,51,52,53]. BHRF1 miRNAs are expressed in the type III latency cells, they inhibit apoptosis and promote passage through the cell cycle [54]. On the other hand, the BART miRNAs are expressed in all types of latency and have been shown to be important for latency maintenance [55].

The second human gammaherpesvirus, KSHV, so far is not known to have multiple latency programs, the way it has been described for EBV. Instead, there are four major open reading frames (ORFs) expressed during KSHV latency: ORF73 (LANA), ORF72 (viral cyclin—vCyc), ORF71 (viral FLICE inhibitory protein—vFLIP), and ORF K12 (Kaposins) (Table 1) [27]. LANA is the origin binding protein of KSHV and is therefore responsible for latent replication of the viral genome as well as tethering the genome to cellular chromosomes [24,56,57]. Additionally, LANA also inhibits the action of p53 [58] promoting the survival of infected cells and inhibits the function of Rb [59]. LANA was shown to promote cell cycle progression by binding and sequestering glycogen synthase kinase-3β (GSK3β) [60]. KSHV vCyc is a homologue of the cellular cyclin D2 [61] and was shown to contribute to the tumorigenic properties of KSHV [62]. vFLIP similarly to cFLIP promotes cell survival, by inhibiting caspase 8. It also inhibits autophagy promoting cell survival [63]. It induces NF-κB signaling and therefore promotes latent state and prevents lytic reactivation [64]. The fourth latent ORF encodes a family of Kaposin proteins (Kaposin A, Kaposin B, and Kaposin C) [65]. Kaposin A is able to induce cell transformation [66] and was reported to inhibit apoptosis through interaction with septin 4 [67]. Kaposin B was shown to stimulate cytokine release and therefore contribute to the proinflammatory environment of KSHV-associated tumors [68]. The function of Kaposin C is not known yet.

In addition to the four major latent KSHV ORFs expressed in the KSHV-associated tumors: PEL (primary effusion lymphoma) and KS (Kaposi’s sarcoma) cells, there is also vIRF3 (also called LANA2), which is expressed only in PEL and multicentric Castleman’s disease (MCD) [69]. This protein is a homologue of the cellular interferon regulatory factor (IRF) and inhibits interferon (IFN) induction and apoptosis [69,70,71]. Another protein expressed at low levels in latent PEL cells, but not in KS is vIL-6, which is a constitutively active homologue of cellular IL-6 [72,73,74,75,76]. vIL-6 promotes proliferation and prevents apoptosis of PEL cells [76,77,78]. The cell type specific expression of vIRF3 and vIL-6 suggests that KSHV might have different latency programs depending on the type of infected cell. In fact this has been observed in a recent study [79] comparing latency in TREx-BCBL1RTA and iSLK.219. Another type of exception to the canonical latency program of the four major latency proteins is the K1 protein, which is expressed in latency at low levels and is induced in the lytic cycle [72,80]. K1 is a membrane protein that mimics B cell receptor signaling. It is able to promote cell growth and transformation [81,82] and prevent apoptosis of infected cells [83,84]. K1 is a functional homologue of EBV LMP-2A. 

Similar to EBV, KSHV also encodes non-coding RNAs, including miRNAs [85,86], long non-coding RNA-polyadenylated nuclear RNA (PAN) expressed at only low levels in latency [87], and circular RNAs encoded in vIRF4 and PAN loci [49]. KSHV miRNAs have been shown to promote angiogenesis [88], affect differentiation of infected cells [89,90], and prevent lytic reactivation through different mechanisms [91,92].

Initially, the latency program for HCMV was thought to include, as in the case of other herpesviruses, a limited number of genes. Therefore, also in the case of this virus several genes associated with latency were identified, including: UL138, UL111A, latency unique nuclear antigen (LUNA), US28, a splice variant of IE1—IE1x4, lncRNA2.7, 4.9, and UL144. UL138 is required for HCMV latency establishment (Table 1) [28,93], it induces TNF receptor type I (TNFRI) [94] and downregulates multidrug resistance-associated protein-1 (MRP1) [95]. Additionally, it was shown to increase surface expression and activate epidermal growth factor receptor (EGFR), a receptor that is critical for cell survival, proliferation, and differentiation and therefore promote virus persistence [96]. LUNA, latency unique nuclear antigen (also called UL81-82ast) is an anti-sense transcript encoded in the UL81–82 locus [93,97,98]. LUNA is required for reactivation [98] and more recently it was published to have de-SUMOylating activity, which allows it to disrupt promyelocytic leukemia (PML) nuclear bodies [99]—cellular structures with an established anti-viral function. US28 is a chemokine receptor (vGPCR)—that has been detected in HCMV latency [93,100,101,102]. It was shown to affect differentiation of infected cells and to be required for latency establishment in myeloid progenitors [102,103,104,105]. US28 affects several signaling pathways, which results in silencing of the HCMV major immediate early promoter (MIEP) [103].

Some of the HCMV proteins show expression of specific splice variants depending on the phase of the viral life cycle. One such example is UL111A, which encodes a viral homolog of IL-10, which is expressed in the lytic cycle, while in latency a shorter splice variant LAcmvIL-10 is detected [106,107]. Cellular IL-10 has broad immunosuppressive properties, but the LAcmvIL-10, so far has only been shown to downregulate MHC II in myeloid cells [108], which results in impaired recognition of latently infected cells by CD4+ T cells [109]. Another protein that shows splice-variant-specific expression in latency is IE1x4. In the lytic cycle, IE1 full length protein is the major immediate early transcript, in latency a shorter variant IE1x4 is expressed and has been shown to be important for latent genome replication and maintenance [28]. Additionally, two long non-coding RNAs are expressed during HCMV latency: RNA2.7 and RNA4.9. The lnc4.9 binds polycomb repressor complex (PRC2) and is responsible for the repression of lytic gene expression. In the lytic cycle, lnc2.7 has anti-apoptotic properties; however, its function in latency has not been studied so far [110]. One more level of complexity in the regulation of latent transcription is demonstrated by UL144, whose expression in latency is virus-isolate-specific [111]. UL144, also called herpesvirus entry mediator (HVEM/TNFRSF14) belongs to the TNF receptor superfamily of proteins and therefore could participate in regulation of immune cell function, but its function in latency still needs to be investigated [112].

All of the HCMV transcripts described above play undoubtedly crucial roles in latency, however, an increasing number of studies show that the expression pattern in latency includes many more genes [93,101,113,114,115]. Rosetto et al. have shown the expression of mRNA of lytic replication genes (UL84 and UL44) in both naturally infected cells from HCMV positive donors and in latent models [113]. Another study which also used both natural and model latency systems has identified an even larger group of genes expressed in latency [114]. The authors classified HCMV genes into two groups. The first one contained highly expressed genes, which were not differentially regulated in latency vs. lytic replication. 30 genes classified to the second group were expressed at overall lower level and were differentially expressed between these two states. This group of genes might be important in defining the distinct infection patterns. Most recent results suggest that in the case of HCMV the difference between the lytic and latent transcriptome might be rather quantitative than qualitative and that the set of viral genes expressed in latency resembles the genes expressed in the late stage of the lytic cycle [115]. Certainly, it is well documented that after initial infection associated with high transcription levels, the HCMV genome becomes chromatinized and progressively repressed, which promotes the establishment of latency characterized by overall lower gene expression [6]. Latent transcription program of HCMV remains a controversial topic and further research will be needed to fully define it.

Most of the human herpesviruses maintain their genomes during latency in an extrachromosomal form of episomes. However, as mentioned earlier, HHV-6A/B represents a unique example of genome integration into telomeric regions of chromosomes [116]. Since HHV-6 has a wide cell tropism, the integration might occur also in gametes (germinal cells). According to Mendelian law, the integrated HHV-6 genome can be transferred to 50% of the offspring. This situation can result in an individual carrying an integrated copy of the HHV-6 genome in all the cells of the body. This condition is called inherited chromosomally integrated HHV-6 (iciHHV-6) and occurs with an approximate frequency of 1% among the world human population [117,118]. 

The transcriptional activity of integrated HHV-6A/B genome has not been studied extensively. In fact, comprehensive annotation of HHV-6A and HHV-6B genomes has been carried out only very recently [119]. Long before this extensive transcriptome and translatome analysis were performed, four latency associated-transcripts of HHV-6 (H6LTs) were described in HHV-6B-latently infected macrophages and these transcripts were suggested to give rise to three proteins: ORF99, ORF142, and ORF145 [120]. However, more sensitive assay revealed that these H6LTs were only detectable during latency establishment and upon reactivation, but not during latency as such [121]. In HHV-6A-infected peripheral blood mononuclear cells expression of U94 transcript was detected during latency. U94 was shown to inhibit viral lytic replication and was therefore proposed to promote latency establishment and maintenance [122,123]. More recent RNA-seq analysis did not detect any transcripts in patient derived iciHHV-6A cells and in in vitro generated 293-HHV-6A cells. These results might suggest that the latent HHV-6 genome is maintained in a transcriptionally quiescent and highly condensed chromatin state confirmed by detection of repressive histone modifications H3K9me3 and H3K27me3 [124]. Future studies will hopefully reveal the reason for differences in detection of latent transcripts in HHV-6A infected cells. Recent publications highlighted the role of small non-coding RNAs (sncRNAs) including miRNAs in herpesvirus latency maintenance and in regulation of the lytic switch [125,126]. In the case of HHV-6, transcription of miR-U86 was detected in latency and observed to be upregulated in the so-called ‘transactivation’, which is the very early stage of lytic reactivation characterized by the expression of some lytic transcripts, but lack of expression of viral proteins [127]. Upregulation of miR-U86 upon transactivation remains in agreement with the previous suggestion that miR-U86 inhibits the expression of U86 and therefore prevents lytic replication and supports latency [128]. Taken together, detection of the H6LTs only during latency establishment and upon reactivation, but not in latency as such [121] and detection of several transcripts including miR-U86 upon transactivation [127], led to the hypothesis that H6LTs and miRNAs could be detected only at specific time points, i.e., at the point of latency establishment and during transactivation [124,127].

Unfortunately, mechanisms of HHV-7 latency establishment and maintenance are still under investigation and latency genes of HHV-7 have not yet been identified.

Initially, HSV latent transcription was considered to be limited to the latency associated transcripts (LATs), which are found on the opposite strand of the ICP0 locus, encoding the major immediate early protein (Table 1) [129,130]. Of note, latency-associated transcripts (LATs) of all alphaherpesviruses originate from genome regions encoding ICP0 homologs [131]. The LATs are not required for latency establishment [132,133,134], but they participate in repression of lytic genes and therefore contribute to latency establishment [135,136,137,138]. LATs also inhibit apoptosis of latently infected cells [139,140,141]. The LAT transcripts include short non-coding RNAs—sRNA1 and sRNA2 [129] and the long non-coding RNAs (lncRNAs) [132]. Out of 17 miRNAs encoded by HSV-1, 11 are found within or close to LAT locus and 7 are expressed in latency [142,143,144,145]. The latently expressed miRNAs repress transcription of lytic genes and affect viral replication in neuronal cells [145,146]. Over the years, evidence has accumulated showing that the latency of HSV-1 might be much less “silent” than previously thought and that expression of lytic genes can be detected in latent cells [147,148,149,150,151,152,153,154,155,156,157,158,159,160]. It was shown that reactivation of HSV in latency is quite frequent and spontaneous [161,162]. It has also been observed that only ~30% of latently infected neurons expresses LATs, but the population of cells that express LATs changes over time, such that each cell expresses LATs at some point [163,164,165,166]. Additionally, ICP0—the lytic protein, was shown to regulate transcription of LATs and chromatin structure of the latent genome [157]. These observations challenge the binary view on HSV transcription, which assumes either highly regulated lytic program or continuous expression of latent genes (LATs) [160,167]. One of the hypotheses explaining the detection of lytic transcripts in latency says that they result from frequent abortive reactivation episodes in only few cells [168]. Alternatively, detection of lytic transcripts in latent models could imply changes (possibly also cyclic changes) in viral gene expression signifying regulation of viral transcription in latency, i.e., a possible “latency program” as others put forward [160,169]. It remains to be determined whether detection of lytic gene expression in latency corresponds to frequent abortive infection or is a result of dynamic expression changes. 

In VZV-latently infected human ganglia, several transcripts have been detected including ORF21, 29, 62, 63, and 66 [170,171]. Further quantitative assays revealed that only ORF63 was detected consistently in latently infected trigeminal ganglia, it was also the most abundant transcript [172,173]. ORF63 was shown to be important for latency establishment and to suppress apoptosis in human ganglia under experimental conditions [174,175]. Subsequently, RNA as well as the protein of an additional gene, ORF4 were identified in latently infected human ganglia and shown to be important for latency establishment [176]. Recently identified VZV latency-associated transcript (VLT) provides a novel aspect and might bring breakthroughs in our understanding of VZV latency and reactivation [177,178]. VLT is expressed in a few copies per neuron during the latent phase, but alternatively spliced VLT variants can be detected during lytic cycle and are termed VLTly. VLT in contrast to VLTly, is possibly expressed from a neuron specific promoter, which would explain a single unique VLT isoform predominating in latently infected ganglia [177]. The VLT resembles the HSV LAT and is encoded antisense to VZV ORF61 (a homolog of HSV ICP0). Both proteins VZV ORF61 and HSV ICP0 (infected cell polypeptide 0) belong to the immediate early category (IE) and are required for the viral lytic cycle. Therefore, it is hypothesized that VLT plays a role in latency maintenance by inhibiting ORF61 expression and function [131,177]. In conclusion, VZV latency in human ganglia is in most cases restricted to the expression of both latency associated transcripts: VLT and ORF63; however, not all of the TG (trigeminal) neurons are transcriptionally active. Despite the potential of both of these transcripts to be translated during the latent phase, the viral proteins remain undetected in latently infected human TG neurons by immunohistochemistry [177].

## 4. Virus Reactivation

Herpesviruses from all the three subfamilies can periodically reactivate to cause symptomatic recurrent infection or be asymptomatically shed to new hosts. Reactivation and re-entering a lytic cycle can be triggered by a broad range of physiological and environmental factors (Table 1). 

Physiological triggers that induce gammaherpesvirus reactivation are not as clear as in the case of other herpesviruses (see below). Infections by other viruses, such as HIV, HSV-1, HSV-2, HHV-6, HHV-7, HCMV, and papillomavirus can contribute to lytic reactivation of KSHV [179,180,181,182]. Further research has shown that activation of Toll-like receptors 6 and 7 (TLR6 and TLR7) by other viruses can trigger KSHV reactivation [180]. Similarly, previous studies suggest that HIV-1, HPV, HHV-6, *Plasmodium falciparum*, Leptospirosis, and Group A Streptococci stimulate EBV reactivation [183,184,185,186,187,188]. In the case of EBV, it was also highlighted that the lytic cycle can be induced by *Treponema pallidum* through TLR2 and B-cell receptor (BCR) activation [189]. The increased number of antigens or decreased level of circulating antibodies are also likely to trigger EBV reactivation in memory B-cells through BCR [190]. Moreover, proinflammatory cytokines (Oncostatin M-OSM, hepatocyte growth factor/scattered factor—HGF/SF and IFNγ) produced in response to infection can facilitate lytic replication of KSHV [180,191,192,193]. In the case of EBV prostaglandin E2 (PGE2) was shown to induce lytic reactivation [194]. Taken together, infection with other viruses or bacteria can induce lytic reactivation of gammaherpesviruses through the activation of Toll-like receptors, BCR as well as cytokines produced during infection. Hypoxia is another possible cofactor of KSHV reactivation since it was found that KS tumors are more likely to appear in body parts that are relatively weakly supplied with blood and oxygen [180,195]. Low oxygen conditions can also facilitate EBV reactivation [196]. Hypoxia inducible factor 1α (HIF1α) can directly induce EBV reactivation by activating the expression of BZLF1, a lytic switch protein [197]. In fact, reactivation of KSHV by hypoxia and proinflammatory cytokines involves oxidative stress and reactive oxygen species (ROS). Hydrogen peroxide can induce the lytic cycle of KSHV through activation of mitogen-activated protein kinase (MAPK) pathways [198]. Upregulated ROS production also induces KSHV reactivation through inhibition of NF-κB pathway [199]. Stress and changes in catecholamines levels can induce EBV reactivation [200]. Elevated levels of stress hormones together with dysregulation in cell-mediated immunity have been implicated in EBV reactivation in astronauts during spaceflight [201,202,203,204]. Exposure of astronauts to unique non-terrestrial stressors—such as variable gravitational forces, cosmic radiation, and microgravity—dysregulates both immune and endocrine systems facilitating herpesvirus reactivation, although it remains asymptomatic in most cases [204]. Other environmental stressors such as chemotherapy and radiotherapy can induce lytic reactivation of EBV, probably due to suppressed immune system [205,206]. 

A variety of chemical and biological factors can induce gammaherpesvirus reactivation in cell culture latency systems. Strong inducers of KSHV and EBV lytic reactivation are the phorbol ester TPA (12-O-tetradecanoylphorbol-13-acetate) that broadly activates signal transduction cascades, as well as two histone deacetylase inhibitors: sodium butyrate (NaBu) and trichostatin A (TSA), [207,208,209,210]. TPA has been used for induction of EBV reactivation with higher efficacy than hypoxia [196]. TPA induces both EBV and KSHV lytic cycle in latently infected cells via MAPK/ERK and protein kinase C (PKC) pathways [208,211]. HDAC inhibitors reduce the overall histone deacetylation and therefore lead to global transcriptional activation. Valproic acid (VPA, 2-propyl-pentanoic acid), another histone deacetylase (HDAC) inhibitor, exerts opposite effects on KSHV and EBV reactivation. It strongly induces KSHV lytic cycle, whereas it inhibits lytic reactivation of EBV. The explanation for these varying outcomes might be different pathways leading to EBV vs KSHV reactivation [209].

The reactivation of viruses belonging to Betaherpesvirinae is mainly mediated by immune response and cytokines that are released and stimulate the terminal differentiation of infected cells. The important reservoirs of latent HCMV are the CD34+ hematopoietic stem cells and CD33+ myeloid progenitors, which develop into latently-infected CD14+ blood monocytes. Latent HCMV can reactivate in these cells as a consequence of differentiation towards macrophages and myeloid dendritic cells (DCs) driven by proinflammatory cytokines (IFNγ, TNFα, IL-4, GM-CSF) (Figure 1) [8,212]. In comparison to neurons that represent a life-long reservoir of latent alphaherpesviruses, the hematopoietic reservoir of latent HCMV is not long-lasting and it rather comprises a temporary stage due to much shorter life span of infected cells [212]. Interestingly, recent study demonstrated that HCMV induces differentiation of hematopoietic progenitor cells (HPCs) into a long-lived and immunosuppressive subpopulation of monocytes to achieve latency [213]. Betaherpesviruses are frequently reactivated in allograft recipients [214,215,216,217]. Immunosuppressive therapy that is applied in these patients to prevent and treat graft rejection and graft versus host disease potently suppresses cellular immunity, making these individuals more prone to viral reactivation [218]. A reduced number of CD8+ T cells that play a crucial role in controlling HCMV latent infection contributes to HCMV reactivation in immunosuppressed patients [219]. In cell culture systems, which model the situation in transplant patients, the presence of allogeneic peripheral blood mononuclear cells (PBMCs) can reactivate HCMV from latently infected monocytes. These data suggest that cytokines released as a result of allogeneic activation of T cells induce the differentiation of monocytes towards macrophages and consequently lead to HCMV lytic reactivation in those cells [214]. Although generally, HCMV reactivation does not comprise a common clinical problem, it might have serious clinical consequences in immunosuppressed patients such as those infected with HIV or undergoing immunosuppressive therapy after transplantation [220]. Alloreactivity is responsible for induction of virus reactivation not only as a result of recognizing foreign (transplanted) cells, but functions also to recognize presence of pathogens. In many cases, infection of cells positive for a herpesvirus with other pathogen induces reactivation of that herpesvirus. Additionally, reactivation of HHV-7 was shown to induce lytic replication of HCMV in allograft recipients [221]. Moreover, HCMV tends to reactivate in astronauts during and after spaceflight due to immune system dysregulation as well as activation of hypothalamic-pituitary-adrenal (HPA) and sympathetic-adrenal-medullary (SAM) axes of endocrine response. Thus, astronauts shedding HCMV might pose a risk to newborns or immunocompromised individuals after landing on Earth [201,202,204,222]. 

Although the most significant site of HCMV persistence is the myeloid lineage, HCMV can also establish latency in other cell types. There are studies confirming HCMV latency establishment in neural lineage, particularly in primitive neural stem cells, therefore suggesting a link to pathology of central nervous system observed in children with congenital CMV disease [10]. Similar to HCMV latency in myeloid lineage, reactivation of HCMV from latently infected neural stem cells seems to be differentiation dependent. However, contradictory results have been reported with regard to the permissivity of cells derived from different stages of neural differentiation (neural progenitor cells, neural precursor cells, and neurons) for the HCMV lytic cycle [10,223,224,225,226]. 

In cultured latently infected peripheral blood monocytes (PBMs), the differentiation-dependent shift of HCMV to the lytic cycle can be induced by granulocyte colony stimulating factor (G-CSF) and hydrocortisone. Although the above mentioned substances were observed to induce the expression of viral immediate early (IE) and early (E) genes, the production of infectious virus was not detected [227]. However, using a protocol for differentiation of CD34+ progenitors to mature dendritic cells (DCs) (involving transforming growth factor-β (TGFβ), TNFα, stem cell factor, granulocyte-macrophage colony-stimulating factor (GM-CSF), and FMS-like tyrosine kinase 3 ligand (Flt-3L) and lipopolysaccharide, it is possible to induce viral particle production [228]. As it was mentioned before, cytokines produced by allogeneic T-cell stimulation can also induce HCMV reactivation in monocytes [214]. Thus, myeloid cell differentiation is essential for HCMV reactivation in both latently infected organisms and cell culture systems. However, the regulation of viral lytic gene expression by the process of differentiation is not fully understood. The chemical compound that is effectively used to reactivate HCMV from latency is the phorbol ester TPA [229]. 

HHV-6A, HHV-6B, and HHV-7 that belong to the Roseolovirus genus of betaherpesviruses share common mechanisms of establishing latency and reactivation. Similar to HCMV, HHV-6 also establishes latency in CD34+ hematopoietic stem cells [9] and in myeloid cell lines [230]. Additionally, under experimental conditions latency can also be established in oligodendrocytes (HHV-6A) and in astrocytes (HHV-6B) [231,232]. Latent HHV-7 is carried in peripheral blood mononuclear cells (PBMCs) and can be reactivated as a result of T-cell activation by anti-CD3 monoclonal antibody and IL-2, as well as anti-CD3 and anti-CD28 monoclonal antibodies. Latent HHV-6B present in PBMCs is resistant to this type of stimulus. However, it can be reactivated by superinfection with HHV-7 [233]. The knowledge about cell types in which HHV-7 can establish latency is limited. 

Among the Roseolovirus genus, latent HHV-6B and HHV-7 present in cultured cells can be reactivated by TPA [234,235], while lytic cycle of HHV-6A in PBMCs can be induced by TPA, hydrocortisone and trichostatin A (TSA) [21]. 

The frequency of clinical reactivation varies among alphaherpesviruses. Symptomatic reactivation of HSV may occur repeatedly and mostly in young people, whereas clinical reactivation of VZV typically occurs once in a lifetime and mostly affects elderly people [236]. The reactivation of HSV and the emergence of cold sores are related to various stimuli. It has been known for over a century that local injury (e.g., surgery) introduced to the nerve results in an incidence of cold sores (re-emergence of herpetic lesions) in the dermatome innervated by the injured nerve [237]. Other signals for reactivation of HSV can be sunlight and UV radiation [238]. It was suggested that immunosuppression and reduced cellular immunity due to UV radiation also contributes to reactivation of VZV, resulting in a higher frequency of zoster outbreaks in the summer [239,240]. Moreover, spacecraft crewmembers can experience VZV reactivation, which is usually asymptomatic and caused by elevated cortisol level together with dysregulation of immune system [201,202,204]. Long duration space mission can also lead to HSV-1 lytic reactivation although less frequently [204]. Changes in core body temperature, such as hyperthermia caused by fever, as well as hypothermia also facilitate the reactivation of HSV [241]. Prostaglandin PGE2 and cytokines such as IL-6 that are produced during high fever can directly stimulate neurons with latent HSV reservoirs [242,243,244]. Persistent psychological stress and fatigue are often responsible for re-emerging herpes symptoms, which partially correlates with reduced CD8+ T cell surveillance of latently infected neurons (Figure 1) [245]. Stressful life events and depression have also been shown to be associated with VZV reactivation (herpes zoster) [246,247,248]. Hormonal changes and hypoxic conditions can also cause HSV reactivation [249,250]. Moreover, it has been observed that in the context of HSV recurrence different triggers can lead to reactivation from different subtypes of neurons [251]. 

Under experimental conditions, several molecular pathways have been targeted to reactivate alphaherpesviruses. Continuous nerve growth factor (NGF) signaling has been known to be essential for maintaining HSV latency in neuron model systems of mice and rats. Thus, withdrawal of NGF from latently infected cultures leads to reactivation of the lytic cycle [252,253]. Inhibition of NGF signaling also induces VZV DNA production in axotomized cadaver ganglia [254]. Recently, it was shown, that NaBu, is able to reactivate alphaherpesviruses in cell culture [255,256]. Taking advantage of naturally occurring mechanism of HSV reactivation mediated by catecholamines and glucocorticosteroids released under influence of emotional stress, dexamethasone, a synthetic corticosteroid is efficiently used to reactivate HSV-1 both ex vivo and in primary neuronal latent systems [257,258]. The treatment of cells latently infected with VZV with phosphoinositide-3-kinase (PI3K) inhibitor (LY7294002) induces viral replicative cycle [256]. 

Host genetic variation, which is a component of infectious disease pathogenesis, has also been found to be associated with frequency of reactivation of herpesviruses. It was suggested that impaired surveillance of latent KSHV infection in individuals from Africa might be associated with 2 HLA-A alleles, A*6801 and A*4301, and 1 HLA-DRB1 allele group, DRB1*04, resulting in enhanced KSHV shedding in saliva [259]. Moreover, single nucleotide polymorphisms (SNPs) in vascular endothelial growth factor A (VEGFA) gene regions have been associated with KSHV viremia in kidney transplant recipients [260]. A long list of host genetic variants underlying control of humoral immune response to EBV has been shown to be associated with an increased lytic reactivation (reviewed in [261]). Similarly, also in the case of HCMV the role of host genetics in immune response to the virus was revealed and a large collection of associated genes was identified eg. TLRs, NKG2D, PD-1, IFNG, IL-6 just to name a few (reviewed in [262]). The clinical course of HSV-1 infection can be modified by MHC class I allotypes (B*18, C*15, and the group of alleles encoding A19), the high-affinity receptor/ligand pair KIR2DL2/HLA-C1, and the CD16A-158V/F dimorphism. Since these polymorphic genes are involved in the adaptive immunity, these findings confirm an important role of CD8+ T cells and NK cells in host defense against HSV-1 infection [263]. Frequency and severity of cold sore episodes due to HSV-1 reactivation can be associated with the haplotype of the Cold Sore Susceptibility Gene-1 (CSSG-1). Individuals with H1, H2, and H4 haplotypes of CSSG-1 experience more frequent and severe herpetic lesions [264]. Genetic variation in the HLA region (HLA Complex P5—HCP5) was linked to susceptibility to herpes zoster resulting from VZV reactivation [265]. 

Greater focus on host genetics of herpesviral infection is needed to predict which individuals are at higher risk of viral reactivation and complications caused by recurrent infection. In the future, larger genomic studies can bring breakthroughs in understanding the host response to herpesviruses.

On the organism level, it is thought that the reactivation of herpesviruses is a combination of both immunosuppression and factors that act directly on latently infected cells. On the cellular level, virus reactivation is believed to be the result of a disturbed ‘balance’ between signaling pathways that activate and inhibit viral lytic replication. Generally, harsh conditions can lead to virus reactivation and progeny production in order to be spread to new hosts. Although certain environmental stimuli are well defined, our knowledge of the exact mechanisms and conditions leading to lytic switch is incomplete. However, the regulation of the latency to lytic switch is essential to develop novel therapeutic strategies that could target the latent human herpesviruses.

## 5. Conclusions

Different herpesviruses establish latency in different cells, which affects the characteristics of the process. The common feature for all herpesviruses is the persistence of the genome in form of an episome, the difference with regards to persistence lies in that the beta- and gammaherpesviruses, encode a protein participating in latent replication and genome tethering, and alphaherpesviruses, do not have such a protein, as they persist in non-dividing neurons. Genomes of all herpesviruses become chromatinized upon entering the cell, therefore transcriptional activity of all of them is subject to regulation by chromatin modifications. 

With regard to gene expression in latency, at first glance each subfamily seems to have its own distinctive pattern (Table 1). Alphaherpesviruses express only LATs or VLTs (for HSV and VZV respectively), the betaherpesviruses have been shown to express a set of latent genes and the EBV, gammaherpesviruses has a whole series of latent expression programs differing in the number of expressed proteins. KSHV so far is not known to have different latency programs, but it was shown that the expression of some latent proteins is cell type specific, so the existence of different transcriptional programs cannot be excluded. Expression of HSV-1 LAT in all of the cells of the population, but only in 30% of the cells at one timepoint, suggests that also alphaherpesviruses might have a latency transcriptional program. Traditionally latency is viewed as a state with limited expression of genes. However, more recent studies show the expression of proteins classified as lytic during latency of viruses belonging to all three subfamilies. Further research is needed to fully understand the phenomenon of lytic gene expression during latency.

Herpesviruses have the ability to reactivate from latency to lytic cycle. Immunosuppression—due to i.e., exposure to UV radiation, stress, or chemotherapy—is a common inducer of the lytic cycle of all herpesviruses. The presence of proinflammatory cytokines is another signal that tends to reactivate herpesviruses. In the case of HCMV the reactivation is observed during the process of myeloid differentiation, which is associated with cytokine secretion. The final differentiation step of memory B cells to plasma cells is also a signal for EBV to enter the lytic cycle. Hypoxia is an important stimulus inducing the lytic cycle in Alphaherpesvirinae (HSV-1) and Gammaherpesvirinae (KSHV, EBV). Finally, infection with another pathogen can also induce reactivation of the latent beta- (HHV-6B) or gammaherpesviruses (KSHV, EBV).

Even though latency looks a bit different in case of each of the subfamilies of the Herpesviridae with increasing number and sophistication of the model systems as well as improved sensitivity of assays the common features of the hallmark of herpesviruses—their latency state—start to emerge (Figure 1 and Table 1).

## Figures and Tables

**Table 1:** Comparison of herpesviral latency reservoirs, latent transcripts, and inducers of lytic reactivation.

Sub Family	Virus	Cell Tropism	Latent Reservoir	Major Latent Transcripts	Inducers of Lytic Cycle
*Alphaherpesvirinae*	HSV1 HSV2	neurons fibroblasts	neurons	LATs: sRNA1 and 2, lncRNAs, miRNAs	immunosuppression, stress, UV hypoxia local tissue trauma hypothermia and hyperthermia cytokines and prostaglandins * hormonal changes NGF withdrawal Dexamethasone, NaBu
	VZV	neurons	neurons	ORF63 VLT	immunosuppression, stress, UV NGF withdrawal PI3K inhibitor, NaBu
*Betaherpesvirinae*	HCMV	fibroblasts PBMCs macrophages dendritic cells endothelial cells	CD34+ HSCs CD33+ myeloid progenitors CD14+ monocytes NSCs	UL138, UL111A LUNA US28 IE1x4 lncRNA2.7 and 4.9 UL144	immunosuppression proinflammatory cytokines * differentiation of cells allogeneic T-cell activation TPA
	HHV-6 HHV-7	T cells	CD34+ HSCs PBMCs	**HHV-6:** H6LTs:ORF99, ORF142, ORF145 U94 miR-U86 **HHV-7:** no data available	immunosuppression infection with other pathogen * (HHV-6B) T-cell activation (HHV-7) TPA (HHV-6A/6B, HHV-7), TSA (HHV-6A)
*Gammaherpesvirinae*	EBV	B cells epithelial cells	B cells	**Latency I:** EBNA1 **Latency II:** EBNA1 and LMP1 and/or 2 **Latency III:** EBNA1, 2, 3a, 3c, 3b, LP and LMP1, 2a and 2b	immunosuppression, stress, chemotherapy, radiotherapy, hypoxia infection with other pathogen * prostaglandins * BCR crosslinking, TPA, NaBu, TSA
	KSHV	B cells endothelial cells	B cells	LANA (ORF73) vCyc (ORF72) vFLIP (ORF71) Kaposins vIRF3 (PEL and MCD) miRNAs	immunosuppression, UV proinflammatory cytokines * hypoxia infection with other pathogen * TPA, NaBu, TSA, VPA

* For more detailed description please see text Section 4. Virus reactivation. HSV—herpes simplex virus; VZV—varicella zoster virus; HCMV—human cytomegalovirus; HHV—human herpesvirus; EBV—Epstein–Barr virus; KSHV—Kaposi sarcoma-associated herpesvirus; PBMCs—peripheral blood mononuclear cells; HSCs—haematopoietic stem cells; NSCs—neural stem cells; NPCs—neural progenitor cells; NGF—nerve growth factor; NaBu—sodium butyrate; PI3K—phosphoinositide 3-kinase; TPA-12-O—tetradecanoylphorbol-13-acetate; TSA—trichostatin A; BCR—B-cell receptor; VPA—valproic acid; LATs—latency associated transcripts; ORF—open reading frame; VLT—VZV latency-associated transcript; LUNA—latency unique nuclear antigen; EBNA—Epstein–Barr nuclear antigen; LMP—latency-associated membrane proteins; LANA—latency-associated nuclear antigen; vFLIP—viral FLICE inhibitory protein; vCyc—viral cyclin; PEL—primary effusion lymphoma; MCD—multicentric Castleman’s disease.
